# CDH3 Mutation in Saudi Arabia: A Case of Hypotrichosis With Juvenile Macular Dystrophy

**DOI:** 10.7759/cureus.106554

**Published:** 2026-04-06

**Authors:** Enam Danish, Rola Alsulami, Hamza Baeshen

**Affiliations:** 1 Department of Ophthalmology, King Fahad Armed Forces Hospital, Jeddah, SAU; 2 Department of Ophthalmology, Makkah Health Cluster, Makkah, SAU; 3 College of Dentistry, Batterjee Medical College, Jeddah, SAU

**Keywords:** cdh3 mutation, hjmd, hypotrichosis, juvenile macular dystrophy, visual impairment

## Abstract

Congenital sparse scalp hair and progressive vision loss are hallmarks of hypotrichosis with juvenile macular dystrophy (HJMD), a rare autosomal recessive condition. We present a rare case of HJMD from Saudi Arabia. A six-year-old Saudi girl, born to first-cousin consanguineous parents, presented with sparse scalp hair growth from birth and decreased night vision from one year of age. Full-field electroretinography (ffERG) suggested cone-rod dysfunction. Fundus photographs showed pigmentary degenerative changes around the macular area and mid-periphery. HJMD was suspected. A homozygous missense mutation, c.1918T>G (p.Cys640Gly), was discovered in exon 4 (NM_001793.5) of the *CDH3* gene by whole-exome sequencing. Patients with these clinical characteristics should be evaluated for HJMD, a rare genetic cause of hypotrichosis and macular degeneration. Although several mutations have been reported in Saudi Arabia, the *CDH3* c.1918T>G variant identified in this patient further expands the understanding of the genetic spectrum of HJMD in the region.

## Introduction

Clinical symptoms of hypotrichosis with juvenile macular dystrophy (HJMD), OMIM 601553, a rare autosomal recessive condition, include inadequate scalp hair development from birth and gradual visual impairment that leads to blindness [1]. Mutations in the *CDH3* gene, which produces the P-cadherin protein and is located on chromosome 16q22.1, are responsible for this disorder [2]. This glycoprotein is essential for adherens junctions in several epithelial tissues, including hair follicles [3] and the retinal pigment epithelium (RPE) [4,5].

Patients with HJMD typically have short, sparse hair that does not regrow after birth. Visual function progressively deteriorates during the first to third decades of life and may ultimately result in blindness. The combination of hypotrichosis and macular pigmentary changes observed on fundus examination forms the basis for the clinical diagnosis [6]. Here, we present a rare case of HJMD in Saudi Arabia.

## Case presentation

History

A six-year-old Saudi girl, born to first-cousin consanguineous parents, presented to the ophthalmology department complaining of decreased night vision from the age of one year. Shortly after her birth, her parents had noticed sparse scalp hair growth. The child is third in birth order, and she was born full term with no pregnancy complications. No siblings had the same complaints, and there was a family history of maternal cousins with alopecia and visual impairment.

General physical examination

**Figure 1:**
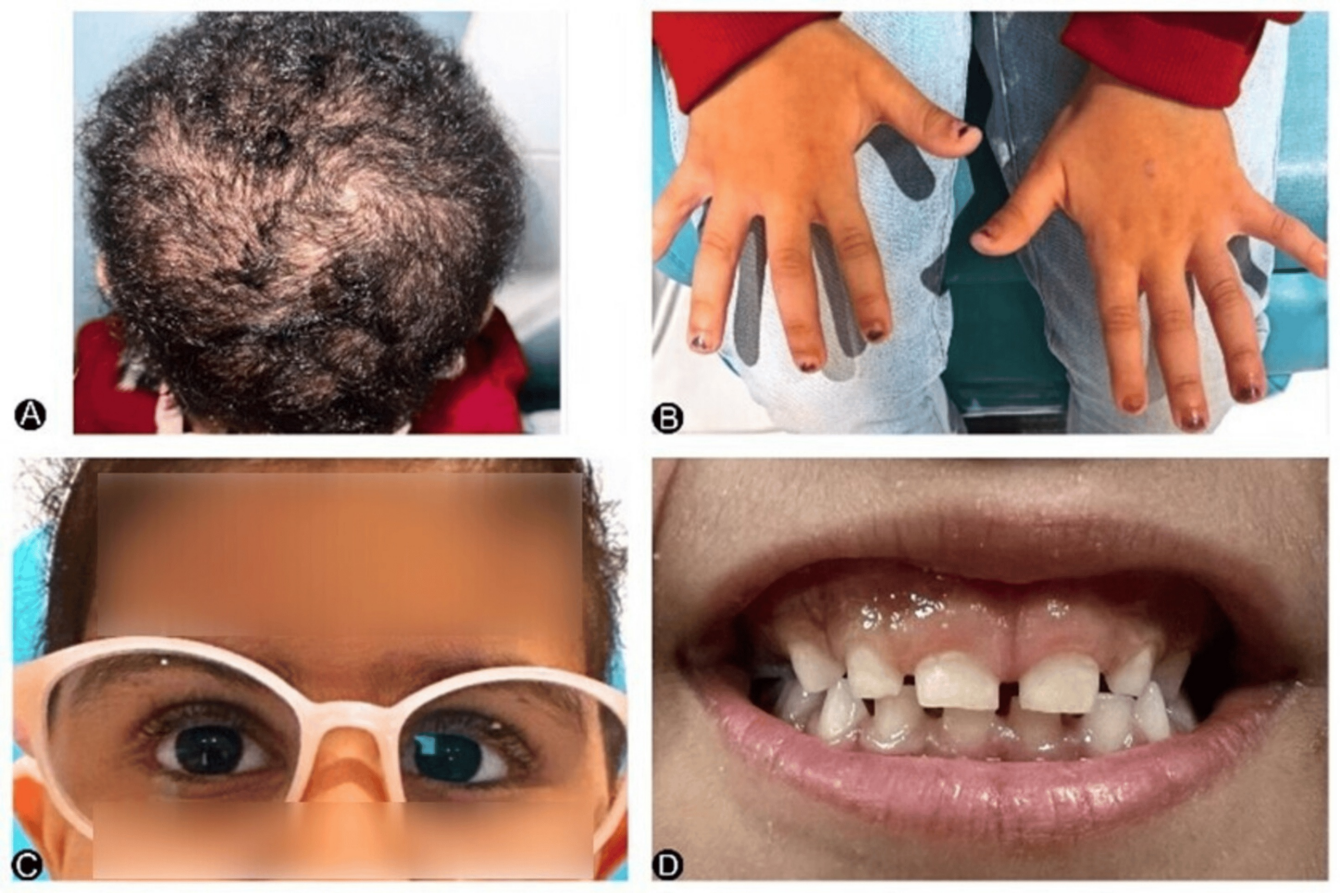
(A) Sparse and short scalp hair. (B) Normal-shaped fingers without deformity. (C) Bilateral moderate hyperopic spectacles. (D) Outside view of the teeth.

On examination, the scalp exhibited diffuse thinning and sparse hair with multiple irregular patches of alopecia. There was no obvious scalp scarring or erythema (Figure 1A) and no dysmorphic features. The skin appeared normal. The palate was intact without clefting. The tongue appeared normal with no structural abnormalities. Bilateral hand examination demonstrated normal-shaped fingers with no evidence of syndactyly, ectrodactyly, or deformities (Figure 1B).

Dental manifestations revealed the congenital absence of upper right first Primary Molar #54 (FDI numbering system) or upper first Primary Molar #D (Universal numbering system). Its predecessor, the upper first premolar tooth bud, was sound and present. All other teeth were present, and gingival pigmentation was observed in this patient (Figure 1D).

Ophthalmologic examination

**Figure 2:**
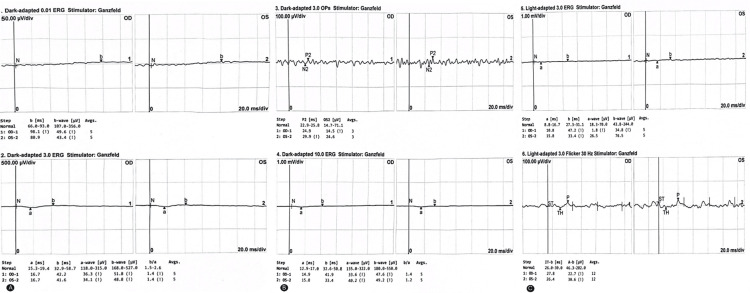
Full-field electroretinography (ffERG) of both eyes. (A) Showing minimal reduced b-wave of rod response in dark-adapted 0.01 stimulus suggesting minimal rod affection. (B) Negative response of b-wave in dark-adapted 10.0 stimulus showing significant cone dysfunction. (C) Reduced b-wave of light-adapted 3.0 flicker stimulus, confirming cone dysfunction.

The pupils were aligned in the primary position with intermittent left eye esotropia of varying degrees. Her visual acuity was 0.6 + 2 in both eyes. Cycloplegic refraction was +4.00 -1.5 × 175 in the right eye and +4.50 -1.5 × 175 in the left eye (Figure 1C). On fundus examination, there was macular retinal pigmented epithelium (RPE) mottling with peripheral hypopigmentation. Full-field electroretinography (ffERG) showed significant cone dysfunction with minimal reduction in rod function, suggesting cone-rod dystrophy (Figure 2A-2C).

**Figure 3:**
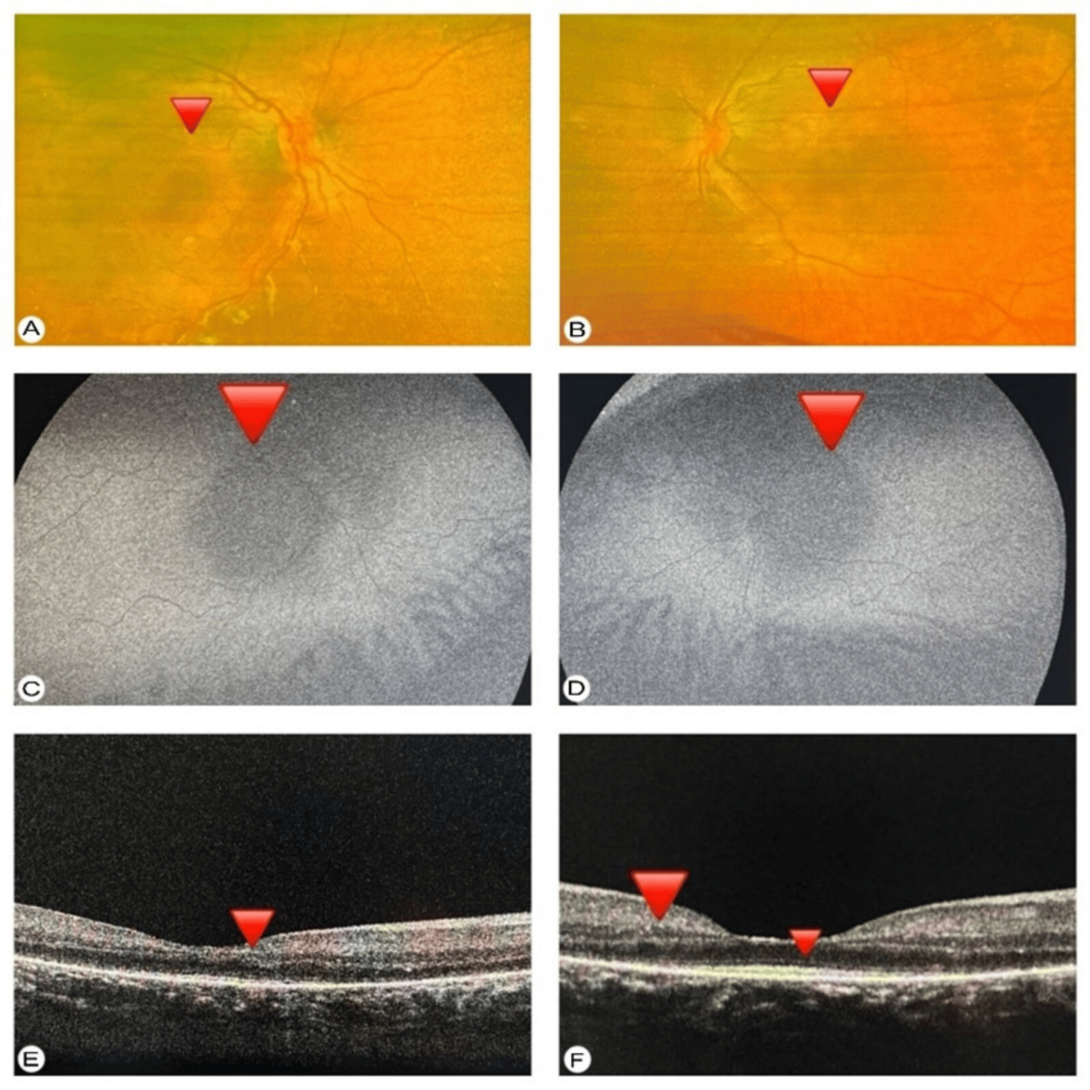
(A, B) Right and left fundus photography showing subtle macular pigmentary changes with ill-defined RPE mottling and hypo-pigmentation. (C, D) FAF showing macular hypo-autofluorescence with surrounding stippled hyper-autofluorescence consistent with RPE disruption and macular dystrophy. (E, F) Spectral-domain OCT showing significant foveal thinning, with disruption of the EZ and attenuation of the RPE (red arrowheads). RPE, retinal pigment epithelium; FAF, fundus autofluorescence; OCT, optical coherence tomography; EZ, ellipsoid zone.

Fundus photography showed pigmentary degenerative changes around the macular area and mid-periphery. These findings are consistent with an early stage of macular dystrophy (Figure 3A, 3B). Fundus autofluorescence (FAF) demonstrated macular hypo-autofluorescence with RPE degeneration and macular atrophy (Figure 3C, 3D). Spectral-domain optical coherence tomography (SD-OCT) showed foveal thinning and destruction of the ellipsoid zone (EZ) in the macular area (Figure 3E, 3F).

Genetic testing

**Figure 4:**
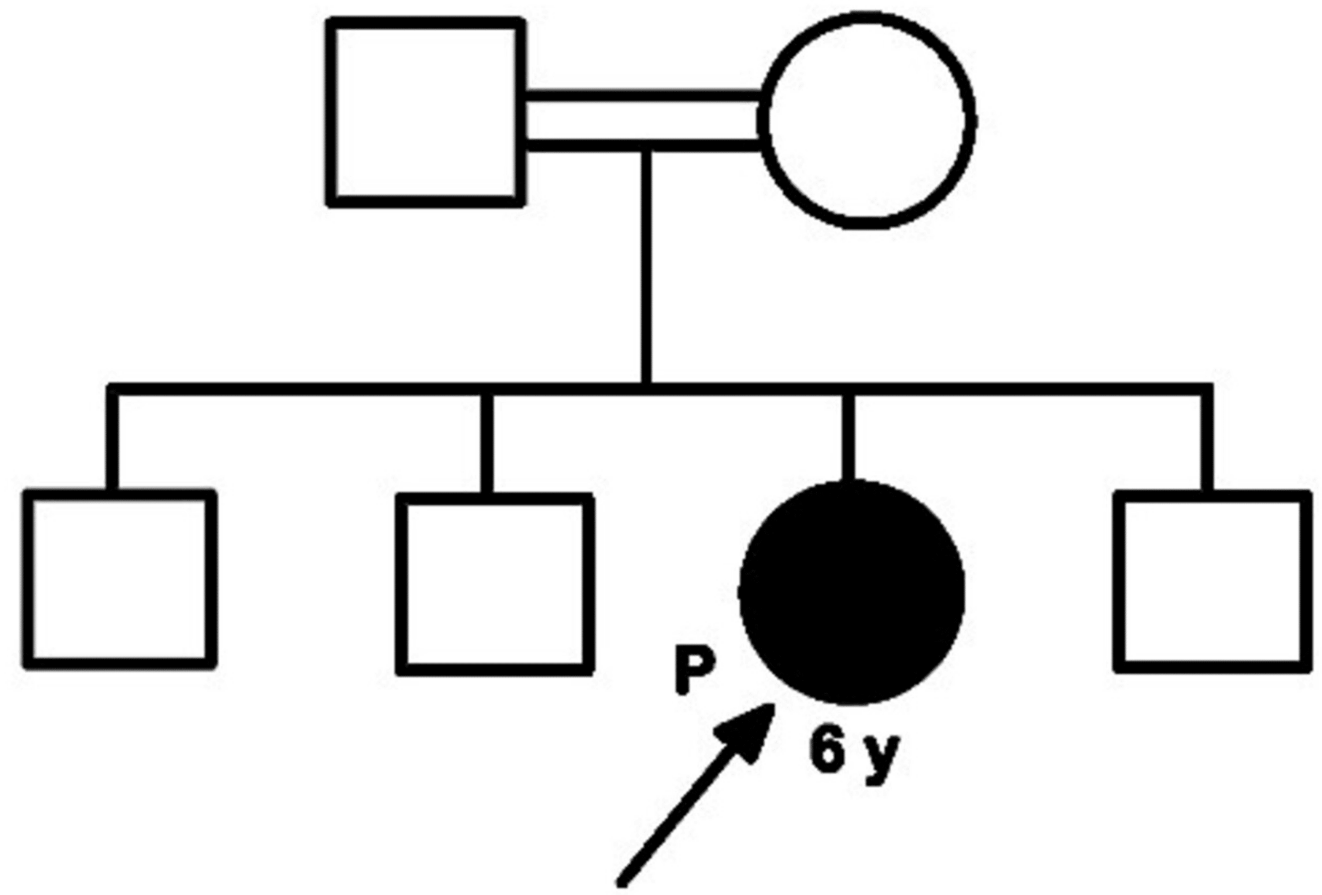
Family’s pedigree.

Through whole-exome sequencing, a homozygous missense variant, c.1918T>G (p.Cys640Gly), was identified in exon 4 (NM_001793.5) of the *CDH3* gene. According to the Centogene diagnostic report, the variant was classified as likely pathogenic. The result was considered consistent with a genetic diagnosis of autosomal recessive CDH3-related disease. No additional clinically relevant variants were detected. The family’s pedigree is displayed in Figure 4.

## Discussion

HJMD is an autosomal recessive disorder that manifests as aberrant scalp hair growth together with juvenile macular dystrophy, which eventually leads to blindness [7].

HJMD, as well as ectodermal dysplasia, ectrodactyly, and macular dystrophy syndrome (EEMS), are rare autosomal recessive illnesses that are characterized by progressive macular dystrophy and hypotrichosis. These disorders can be brought on by pathogenic mutations in the Cadherin 3 (*CDH3*) gene. P-cadherin, a calcium-binding protein required for intercellular adhesion, is expressed in both hair follicles and RPE cells and is encoded by the *CDH3* gene [8].

A case of *CDH3*-related HJMD in Jordan was reported by Al Zubi et al. [8]. In exon 7 of the *CDH3* gene, the patient carried a homozygous frameshift deletion (p.Gly277AlafsTer20). A 13-year-old girl was reported to have HJMD due to the *CDH3* gene mutation in Turkey [9]. The patient’s vision deteriorated gradually on both sides, accompanied by significant hair loss. Upon fundus inspection, the RPE had bilateral ring-shaped atrophy and patchy intraretinal pigment clumping at the posterior pole. A unique homozygous deletion in exon 5 of the *CDH3* gene (c.447_467del (p.149_156del)) was found in this patient, according to genetic testing.

In Pakistan, two consanguineous families were presented with HJMD transmitted through autosomal recessive inheritance. The *CDH3* gene in the first family has a new homozygous in-frame deletion (c.1308_1310delACT, p.255delTyr), which was discovered by Sanger DNA sequencing and microsatellite marker genotyping. In the other family, a novel homozygous nonsense variant (c.1454G>A; p.Trp485*) in the same *CDH3* gene was discovered using exome sequencing and genotyping using the Affymetrix Gene Chip Genome-Wide Human SNP 250K array. Both novel variants were associated with the illness phenotype in their respective families [10].

Few cases have been recorded from Saudi Arabia [11-13], with the majority being consanguineous relatives. By identifying homozygous recessive *CDH3* mutations (c.307C>T; p.R103 in two sisters and c.1859_1862delCTCT in two unrelated men, ages 5, 13, 17, and 26, respectively), Khan and Bolz found that four individuals from three consanguineous families were affected [11]. Polygonal pigment clumping and a confined core maculopathy, whose margins frequently extended beyond the major arcades, were seen in all patients. One patient had significant central macular atrophy, and a macular hole after an ERG assessment revealed cone-rod retinal dysfunction instead of macular.

Hasanain et al. [12] described a case of *CDH3* mutation related to ectodermal dysplasia and hair anomalies. Examining the hair revealed that it was lusterless, short, and thin, with just a few plaques and alopecic areas.

In both eyes, the macula had parapapillary retinal pigmentation, retinal and choroidal degeneration, and no loss of the retinal nerve fiber layer. Trichorrhexis nodosa was discovered via a hair biopsy. A positive homozygous pathogenic variant in the *CDH3* gene was found by the genetic test. In this instance, the exact gene variant sequence was not specified. Ahmed et al. [13] reported another example of a 40-year-old male born to consanguineous parents who had considerable eye impairment and hair loss from birth. Through whole-exome sequencing, the homozygous stop gain mutation c.C307T (p.Arg103Ter) was identified in exon 4 (NM_001793) of the *CDH3* gene. Following the identification of an incidental/secondary sequence variant, a homozygous missense mutation was found in exon 6 of *ARL2BP* (c.G485A; p.Arg162Gln) and a heterozygous missense mutation in exon 2 of the *TEK* gene (c.G314A; p.Arg105Gln).

The c.1918T>G *CDH3* gene variant was not discovered in any of the previously reported cases. However, the National Center for Biotechnology Information issued information about this gene mutation in the National Library of Medicine. It was reported as a single-nucleotide variant located at 16q22.1, with the genomic locations Chr16: 68691842 (on assembly GRCh38) and Chr16: 68725745 (on Assembly GRCh37) [14].

Although several mutations have been found in Saudi Arabia, the *CDH3* c.1918T>G gene variant revealed in the presented case has not been described in Saudi cases that have been published before. This finding significantly expands our knowledge of the genetic landscape of HJMD in the Kingdom.

To date, the *CDH3* gene is the only known cause of HJMD, a very rare disease. This makes clinical and genetic diagnosis challenging. However, when all clinical features are considered, they should lead to a clinical diagnosis and the specific genetic test to be conducted [15]. As a result, correct diagnosis is critical because cell and gene therapies are being tested in retinal dystrophies [16].

## Conclusions

HJMD is a rare autosomal recessive disorder characterized by progressive visual impairment and sparse scalp hair growth. While several mutations have been documented in Saudi Arabia, the *CDH3* gene variant c.1918T>G identified in this patient contributes to the knowledge of the genetic landscape of HJMD in Saudi Arabia. This emphasizes the importance of genetic testing in clinically suspected patients, particularly in populations with high rates of consanguinity, and may contribute to improved genetic counseling and future therapeutic strategies.
